# Pure Water

**Published:** 1885-02

**Authors:** 


### PURE WATER.

One of the chief agents in transferring disease germs to the human system is impure drinking water.

It is safe to suspect the water of any open well of containing impurities which are detrimental to health, unless a chemical analysis proves that the water is fit for drinking.

The reason for this is very simple. Open wells are built with stone or brick sides, both of which, being porous, allow the moisture in the surrounding soil, above the level of the water in the well, to trickle through the joints. Now, as the earth is filled with underground passages, through which the water flows, somewhat like the blood in the veins of an animal, the cutting off of these passages, by digging a well, leaves their ends open, and the water naturally drains out of them.

Meeting a porous wall, the water percolates that and enters the well. Consequently there is an excellently drained area surrounding the well for a distance of a hundred feet or more. If, in this area, there is a leaky drain, or a cesspool, then that also empties into the well, with what results may be imagined.

There are two kinds of wells, that are, as a rule, safe.

The first is the driven well, for in that there is no open space, and, of course, no inclination for surrounding water to drain into it. The pipe is, at all times, filled with water, and its lower and open end enters the vein from which the supply is taken. These veins are usually streaks of coarse gravel, that are surrounded by a compact, hard layer of clay, which is impervious to water.

The other kind of well is made by digging, in the usual way, but, instead of bricking up the sides, they are built of drain pipe, of, say, ten or twelve inches in diameter, the joints being cemented, and the earth well packed around the pipe. This makes an open well, but no drainage can enter it because the sides are water-tight.

A pump or bucket may be used in it.

Where there is cause to suspect drinking water of containing impurities of an organic nature, a simple test may be made by putting into a glass of the water a drop of a solution of permanganate of potash. This will stain the water a delicate pink color. Let the color be just perceptible. Upon standing for an hour, if the color fades away, do not use the water. In making the test have the glass *perfectly clean*, and stir the solution with nothing but a glass rod.

All city water coming from reservoirs contains organic impurities, but they are generally not of an injurious character, being merely "pond life."

A safe precaution is to boil all drinking water. This will destroy any organic germs it may contain. It must, however, be said that the cholera microbe is believed to be able to stand a much higher heat without being destroyed.

